# Cardiac Arrest during Transesophageal Echocardiogram (TEE) due to Acute Right Ventricular Failure

**DOI:** 10.1155/2021/7427127

**Published:** 2021-12-24

**Authors:** Cooper B. Kersey, Fitsumberhan Medhane, Andrew M. Pattock, Linda Liu, Gary Huang, Abhijit V. Lele, Younghoon Kwon

**Affiliations:** ^1^University of Washington, Department of Medicine, USA; ^2^University of Washington, Division of Cardiology, USA; ^3^University of Washington, Department of Anesthesiology and Pain Medicine, USA

## Abstract

The case of a patient who suffered cardiac arrest while undergoing transesophageal echocardiography (TEE) is presented here. A 75-year-old man with moderate right ventricular (RV) dysfunction and pulmonary hypertension became bradycardic and hypotensive after receiving propofol for procedural sedation. His profound hypotension ultimately led to a pulseless electrical activity (PEA) cardiac arrest. TEE images captured immediately prior to cardiac arrest show a severely dilated and hypokinetic RV, consistent with acute right ventricular failure. This case highlights the potentially fatal consequences of procedural sedation in patients with RV dysfunction and pulmonary hypertension.

## 1. Introduction

The sedation administered during transesophageal echocardiography (TEE) can have profound effects on intraprocedural hemodynamics, particularly in patients with underlying right ventricular (RV) dysfunction or pulmonary hypertension. Current echocardiographic practice guidelines offer no specific recommendations on whether the performing cardiologist or an anesthesiologist should provide sedation for TEE, which can lead to variable types and levels of sedation during procedures [1]. This report demonstrates a case of cardiac arrest during TEE in the setting of RV dysfunction and pulmonary hypertension and briefly reviews TEE-related complications and fatality rates reported in the literature.

## 2. Case Description

The patient was a 75-year-old man with a body mass index of 23.4 kg/m^2^ and an American Society of Anesthesiologists (ASA) class of three. He had a history of end-stage renal disease on hemodialysis and was initially transferred to our hospital from an outside facility for subacute functional decline and encephalopathy. His admission work-up was notable for a leukocytosis of 12,800 cells/*μ*L, *Enterococcus faecalis* bacteremia, and bilateral pleural effusions. His 12-lead electrocardiogram showed sinus rhythm with first-degree atrioventricular block and a right bundle branch block. The patient was started on broad spectrum antibiotics for bacteremia and later narrowed to ampicillin. Repeat blood cultures were negative. On hospital day one, the patient underwent a transthoracic echocardiogram which demonstrated a left ventricular ejection fraction of 45-50% with global systolic dysfunction, moderate RV dysfunction, mild pulmonary hypertension with a pulmonary artery systolic pressure (PASP) of 41 mmHg, and no visible valvular vegetations.

The patient underwent thoracentesis of the right sided pleural effusion, and bacterial cultures of the pleural fluid grew *Enterococcus Faecalis*. Given this multifocal enterococcus infection, TEE was pursued.

On hospital day nine, the patient underwent TEE under monitored anesthesia care. The anesthesia service was consulted due to pulmonary hypertension as per the institutional guideline. The patient was connected to standard monitoring as recommended by the ASA. Supplemental oxygen was provided by a face mask, with end-tidal carbon dioxide monitoring. The patient received a total of 70 mg of propofol administered over the first ten minutes of the procedure in boluses of 10 mg-20 mg. Hemodynamically, the patient's blood pressure at the beginning of the procedure was 140/50 mmHg with a pulse in the low 60 s. His blood pressure dropped precipitously after receiving propofol down to 60/40 mmHg fifteen minutes into the procedure. At that point, the patient became bradycardic and went into a PEA cardiac arrest. The TEE probe was withdrawn, and chest compressions were initiated under the advanced cardiac life support algorithm. The patient was intubated, and return of spontaneous circulation (ROSC) was achieved after four rounds of chest compressions and epinephrine administration. The patient's cardiac rhythm then deteriorated into ventricular fibrillation requiring defibrillation and further chest compressions. ROSC was achieved once again, and the patient was transferred to the intensive care unit. Upon review of the images captured by TEE at the onset of PEA arrest, it was noted that the RV was severely dilated with minimal contractile function, and a small pericardial effusion was found (Video 1). Due to the patient's critical condition, his family decided to transition him to comfort measures only.

## 3. Discussion

Cardiovascular complications from TEE have been documented to occur in 0.08% of cases and cardiac arrest in 1 : 10,000 cases [2, 3] (Table 1). The sedation administered during TEE poses risks particularly to patients with poor cardiac reserve who are sensitive to minor changes in hemodynamics. Determining the appropriate depth of sedation is made difficult by the trade-off between the level of sedation and the patient's tolerance of the TEE probe. If a patient is not sufficiently sedated, they are more likely to have a gag reflex and vagal response to intubation of the TEE probe. An excessive vagal response to the TEE probes can lead to profound vasodilation, systemic hypotension, and a decreased systemic venous return to the right ventricle [4, 5]. On the other hand, patients who are more deeply sedated tend to be more tolerant of the TEE probe but are at higher risk of the adverse hemodynamic and respiratory suppressant effects of the anesthetic agents [2, 4]. One meta-analysis of TEE complications found that hypoxemia (defined as oxygen saturation less than 90%) occurred in 18% of cases [2]. Transient hypoxemia induces pulmonary vasoconstriction, which increases RV afterload and diminishes RV stroke volume [6].

**Figure 1:**
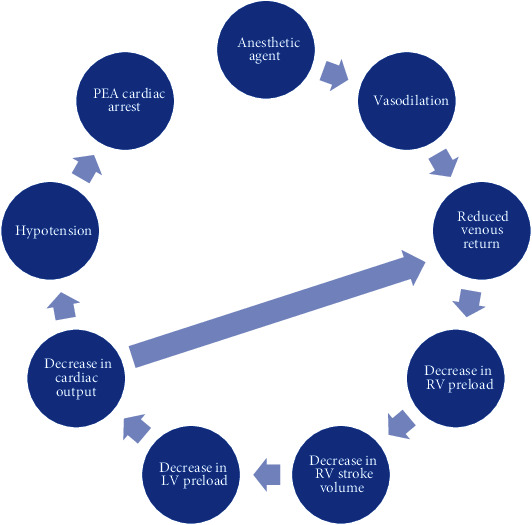
A visual representation of the vicious cycle of right ventricular failure that the vasodilatory effects of anesthetic agents can precipitate [15].

In this case, we suspect that the vasodilatory effects of the propofol led to a profound decrease in RV preload and hypotension. These hemodynamic changes were likely compounded by vagal stimulation of the TEE probe which further contributed to vasodilation and the reduction of RV preload. In a patient with moderate RV dysfunction and pulmonary hypertension, this likely triggered acute RV failure resulting in a PEA arrest (Figure 1). The ideal sedative for a short, minimally invasive procedure like TEE has a quick time to onset, minimal hemodynamic side effects, and a rapid recovery period [7]. Midazolam, fentanyl, ketamine, and propofol are common agents chosen for TEE. However, none of these agents possess all of the characteristics of an ideal sedative agent.

The advantages of using ketamine over propofol include preservation of airway reflexes, maintenance of spontaneous respirations, and sympathetic stimulation, which in turn may allow maintenance of cardiovascular stability. Disadvantages include increased oropharyngeal secretions and neurological side effects such as hallucinations [8]. Propofol, on the other hand, may significantly reduce sympathetic nerve activity and norepinephrine levels at different depths of sedation. These effects result in significant decreases in mean blood pressure of 9% and 18% at moderate and deep sedation, respectively, even in healthy volunteers. Additionally, propofol also reduces reflexive increases in sympathetic nerve activity [9]. Midazolam or fentanyl alone may be insufficient to provide adequate depth of anesthesia. The prevalence of dexmedetomidine use for TEE is unknown, but dexmedetomidine may cause a reduction in pharyngeal muscular tone and respiratory depression similar to propofol, in addition to bradycardia. Dexmedetomidine has been shown to provide satisfactory sedation levels, hemodynamic stability, short recovery time, and acceptable patient and practitioner satisfaction during TEE [10, 11].

In addition to understanding the cardiovascular side effects of sedatives, the proceduralist should also be aware of the risks of hypoxia and hypercarbia in precipitating RV dysfunction. Transient hypoxemia may be sufficient to initiate a vicious cycle of RV dysfunction. Similarly, in patients with a native airway undergoing sedation, hypercarbia is also likely not accurately measured despite the use of end-tidal carbon dioxide monitoring.

We present this case to raise awareness of the risks associated with deep sedation during TEE and illustrate the lack of society and institutional guidelines regarding the choice of sedative agents. The ASA score is a validated measure of patients' intraprocedural risk and could be used to predict which patients undergoing TEE are more susceptible to the hemodynamic consequences of general anesthesia [12]. More research into what patient and procedural factors lead to patient morbidity and mortality during TEE is required to inform practice guidelines and ensure patient safety. Additionally, this case would have been an opportunity to implement TEE-guided CPR, which has been shown to be an efficacious way of monitoring resuscitation efforts in the emergency room setting [13].

Based on this case and available literature, we propose that preprocedural evaluation of patients undergoing TEE includes a detailed review of any contemporaneous TTE data. Data suggestive of pulmonary hypertension with or without RV dysfunction should significantly raise the concern for adverse procedural events. The risk of hypotension, hypoxemia, and hypercarbia need to be balanced with the expectation of TEE tolerance and safety, and this should be discussed between the anesthesiologist and cardiologist as well as during the informed consent process. Monitored anesthesia care or general anesthesia with an endotracheally intubated patient may carry unique risks and benefits and thus should be individualized to the patient and the procedure. In patients identified to be at high risk of adverse events from the hemodynamic effects of propofol, topical anesthetics, ketamine, or midazolam may be useful adjuncts. Alternatively, non-propofol-based anesthesia, such as with dexmedetomidine, may be suitable for certain patients. Lastly, the proceduralist must be familiar with the terminology related to monitored anesthesia care, conscious sedation, and general anesthesia. In any given patient, the depth of anesthesia may vary significantly from light sedation to general anesthesia [14].

This case demonstrates the potentially fatal consequences of procedural sedation in patients with RV dysfunction and pulmonary hypertension. Clinicians should be cognizant of the hemodynamic effects of sedative agents, and institutions should work to create evidence-based criteria to decide if patients undergoing TEE will receive moderate sedation, monitored anesthesia care, or general anesthesia.

## Figures and Tables

**Table 1:** The rates of common complications of TEE documented in the literature from a multicenter retrospective review of over 10,000 patients undergoing TEE (Daniel et al.) and a meta-analysis (Côté et al.).

Complication	Frequency	Source of data
Nonsustained ventricular tachycardia	0.03%	Daniel et al. [3]
Transient atrial fibrillation	0.03%	Daniel et al. [3]
Third degree heart block	0.01%	Daniel et al. [3]
Angina	0.01%	Daniel et al. [3]
Cardiac arrest	0.01%	Daniel et al. [3]
Hypoxemia (SpO_2_ < 90%)	18%	Côté et al. [2]
Bronchospasm	0.5%	Daniel et al. [3]
Mortality	0.1%	Daniel et al. [3]
Retching	39%	Côté et al. [2]
Esophageal perforation	0.1%	Côté et al. [2]

## Data Availability

There are no datasets included in this manuscript.

## Supplementary Materials

Supplementary Materials Video 1: transgastric view of the severely dilated right ventricle with minimal contractile function captured just before cardiac arrest.
