# Supplementary material for: Cardiac Arrest during Transesophageal Echocardiogram (TEE) due to Acute Right Ventricular Failure
Source: Case Rep Cardiol. 2021 Dec 24;2021:7427127. doi: 10.1155/2021/7427127 (PMC8719982; doi:10.1155/2021/7427127)
Supplement: Supplementary Materials — Video 1: transgastric view of the severely dilated right ventricle with minimal contractile function captured just before cardiac arrest. [file 7427127.f1.docx]

**Supplemental Material:**

**Link to Video 1:**

<https://drive.google.com/drive/folders/1TPJfZmtvaZNOjh55ATJqO5S-XefGT3Yo?usp=sharing>

(link to the supplemental material)
